# Maximum Standardized Uptake Value (SUV_max_) of Primary Tumor Predicts Occult Neck Metastasis in Oral Cancer

**DOI:** 10.1038/s41598-018-30111-7

**Published:** 2018-08-07

**Authors:** Grégoire B. Morand, Domenic G. Vital, Ken Kudura, Jonas Werner, Sandro. J. Stoeckli, Gerhard F. Huber, Martin W. Huellner

**Affiliations:** 1 Department of Otorhinolaryngology - Head and Neck Surgery, University Hospital Zurich, Zurich, Switzerland; 2 Department of Nuclear Medicine, University Hospital Zurich, Zurich, Switzerland; 3 Department of Otorhinolaryngology, Head and Neck Surgery, Kantonsspital St. Gallen, St. Gallen, Switzerland

## Abstract

The aim of this study was to investigate the predictability of occult lymph node metastasis using maximum standardized uptake value (SUV_max_) in the primary tumor on pre-treatment 18-fluorodeoxyglucose positron emission tomography FDG-PET in oral squamous cell carcinoma (OSCC) patients who were clinically node negative (cN0) before surgery. A retrospective analysis of all patients treated at the University Hospital Zurich from 2007 to 2016 for OSCC with available pre-therapeutic FDG-PET was performed. We assessed the correlation of SUV_max_ of the primary tumors with the presence of occult nodal disease in the neck dissection specimen (pN+). The study included a total of 71 patients. In the nodal negative group (cN0/pN0), the median SUV_max_ of primary tumors was 9.0 (interquartile range (IQR) 7.4–13.9), while it was 11.4 (IQR 9.9–15.7) in the occult metastatic group (cN0/pN+). The difference was statistically significant (independent samples median test, *P* = 0.037). In a multivariable model, the only independent predictor of occult metastatic disease for cN0 patients was a SUV_max_ ≥ 9.5 (*P* = 0.028). Further, primary tumors with SUV_max_ ≥ 9.5 had a significantly higher risk of local recurrence (Log rank test, *P* = 0.020). In conclusion, we showed that higher SUV_max_ (≥9.5) of the primary tumor is associated with higher occurrence of occult metastatic nodal disease and worse local survival. High SUV_max_ of the primary tumor may encourage clinicians towards more aggressive treatment.

## Introduction

Oral squamous cell carcinoma (OSCC) is an aggressive malignancy characterized by local invasiveness and high propensity to lymph node metastases^1,2^. The presence of neck metastases is the single most important prognostic factor in OSCC, with a decrease of survival of around 50%^3^. Accurate assessment of the nodal status is of utmost importance for prognosis and therapy decision^4–6^. While the management of the neck in nodal positive OSCC is clearly defined, it is more debatable in OSCC presenting without clinically detectable neck disease^5^. Current standard of care are elective neck dissection or sentinel lymph node biopsy, as previously reported^4,7^.

Detection of lymph node metastasis has been improved by modern imaging technologies such a 18F-fluorodeoxyglucose (FDG) positron emission tomography with computed tomography or magnetic resonance (FDG-PET/CT or FDG-PET/MR) imaging^8–10^. The reported negative predictive value of FDG-PET/CT has been reported to approach 90% for cervical lymph nodes in a recent meta-analysis^11^. Nevertheless, a subset of patients with negative FDG-PET/CT will harbour occult metastases that are discovered upon histopathological examination.

Several clinical parameters have been proposed to increase the predictability of occult lymph node metastasis^12^, such as depth of invasion (DOI) or immunohistochemical staining e.g. for CD44 (Morand *et al*., Head Neck, submitted), podoplanin^13^, E-cadherin^14^^.^ as previously reported by our group and others^15,16^. While DOI is measurable on conventional CT or MR images, FDG-PET provides further diagnostic information on tumor metabolism. Such parameters include the maximum standardized uptake value (SUV_max_), metabolic tumor volume (MTV), and/or total lesion glycolysis (TLG). They have been reported e.g. in lung cancer to correlate with the occurrence of occult lymph node metastasis^17,18^. In OSCC, the literature is scarce and it is presently unknown if metabolic parameters derived from FDG-PET/CT can predict occult neck disease.

We therefore conducted a study at our institution with all OSCC patients with available pre-therapeutic FDG-PET/CT or FDG-PET/MR imaging that underwent wide local excision and neck dissection. We assessed the correlation between metabolic parameters derived from FDG-PET/CT or FDG-PET/MR metabolic parameters and occult neck disease.

## Materials and Methods

### Study population

The experimental protocol of the study was approved by the local ethics review board, namely the *Kantonale Ethikkomission Zürich* (protocol number 2016–01799). Study methods were carried out in accordance with the relevant guidelines and regulations. Informed consent was obtained from all subjects. The charts of consecutive histologically proven oral squamous cell carcinoma (OSCC) patients were retrospectively assessed. Patients were treated from 2007 to 2016 with available pre-therapeutic FDG-PET/CT or FDG-PET/MR at the Department of Otorhinolaryngology – Head and Neck Surgery of the Zurich University Hospital, Switzerland. According to our institutional policy, patients with primary tumors staged clinically as either ≥T3 and/or ≥N2b undergo pre-therapeutic FDG-PET due to the elevated risk of distant metastases. Therefore, this study included mainly patients with advanced OSCC stages. However, we also included patients with <T3 or <N2b referred externally with an already available FDG-PET or patients with cT3 category being down-staged on final pathology (pT2). After presentation and discussion at the local interdisciplinary tumor board, patients were consented for wide local excision, neck dissection and reconstruction as needed. All patients were treated with curative intent and no patient had distant metastatic disease at the time of initial diagnosis. Patient with previous head and neck cancer were excluded. Adjuvant radio(chemo)therapy was administered after review of final pathology according to the NCCN Guidelines^19^.

Detailed data on age, gender, smoking, drinking habits, clinical and pathological stage, depth of invasion (DOI), number of nodes dissected, number of positive nodes, local and regional recurrence, distant metastasis, disease-specific survival and overall survival was obtained. Patients were staged according to the Union Internationale Contre le Cancer (UICC), TNM Staging for head and neck cancer, 7^th^ edition 2010^20^. We chose to use the 7^th^ edition and not re-stage all patients with the UICC 8^th^ edition, as the clinical decisions (imaging, surgery, adjuvant treatment) were based on the 7^th^ edition of the TNM staging, which was in use at the time of the patients’ treatment.

Positive and negative clinical neck disease (cN+/cN0) were defined as presence or absence of nodal disease upon clinical examination respectively, that is by clinical exam, FDG-PET imaging, and/or ultrasonography of the neck combined with fine needle aspiration biopsy. Positive and negative pathological neck disease (pN+/pN0) were defined as presence and respectively absence of nodal disease upon histopathological examination of the surgical specimen of the neck. The study cohort was divided accordingly into three groups as follows: the first group was nodal positive, that is with positive clinical neck disease and positive pathological neck disease (cN+/pN+), the second group was nodal negative, that is with negative clinical neck disease and negative pathological neck disease (cN0/pN0), and the third group was occult metastatic, that is negative clinical neck disease but positive pathological neck disease (cN0/pN+).

### FDG-PET/CT or FDG-PET/MR image acquisition

All patients fasted for at least 4 hours prior to the scan. Patients were injected with a standardized dose of 3.5 MBq of FDG per kg body weight (PET/CT) or 3.0 MBq FDG per kg body weight (PET/MR). All patients had a blood glucose level below 10 mmol/l before imaging. During the uptake time of 1 hour, patients rested in a silent, warm and dimmed environment. Scans were acquired using an integrated PET/CT scanner (Discovery VCT or Discovery 690 GE Healthcare, Waukesha, WI) or an integrated PET/MR scanner (Signa PET/MR, GE Healthcare). Scans included either a diagnostic CT scan of the neck after administration of iodinated contrast medium, or a diagnostic regionalized PET/MR scan of the neck using gadolinium-based contrast medium^21^. Detailed technical acquisition protocols have been published previously^8,21^.

### Metabolic parameters

The standardized uptake value (SUV) was calculated automatically (activity in volume of interest (VOI)/(injected dose*body weight)). The SUV_max_ was defined as the hottest voxel within the VOI. SUV_mean_ was defined as the average SUV of voxels within the VOI exceeding 42% of the SUV_max_. The metabolic tumor volume (MTV) was defined as the sum of the volume of voxels with an SUV exceeding a threshold of 42% of the SUV_max_ within the VOI. Total lesion glycolysis (TLG) was defined mathematically as MTV x SUV_mean_. For analysis of FDG uptake, correct placement of volumes of interest on PET images was ensured by side-by-side reading of the corresponding CT or MR images. A written radiological report by board certified nuclear medicine physician/radiologist was available for all FDG-PET/CT or FDG-PET/MR images.

### Statistical analysis

For continuous variables, mean, standard error of mean (±SEM), median, or interquartile range (IQR) are given. To compare distribution among samples, the non-parametric median test was used for continuous variables. Binary variables were associated in contingency tables using the two-tailed Fisher exact test. Correlations between continuous variables were assessed using the two-tailed Spearman rho test. Curve estimations were performed using a linear model not including a constant in the equation. A binary logistic regression model was built to assess the factors predictive of occult lymph node metastasis for all cN0 patients, including all relevant factors in the multivariable model. Survival curves were built according to Kaplan-Meier and the log-rank test was used to compare factors. A *P* value lower than 0.05 was considered to indicate statistical significance. Statistical analyses were performed using SPSS® 23.0.0.0 software (IBM®, Armonk, NY, USA)^22^.

## Results

### Patient and Tumor Characteristics

A total of 71 consecutive patients were included in the study. The baseline characteristics according to study group are shown in Table 1. In total, 37 (52.1%) patients received adjuvant radiotherapy, 11 (15.5%) patients concomitant chemotherapy and 3 (4.2%) patients concomitant Cetuximab. Mean follow-up for the cohort was 20.5 months (±SEM 2.24).

**Table 1:** Baseline Characteristics for Study Cohort and for Study Groups.

Variable	All patients N = 71	Nodal positive cN+/pN+ N = 24	Nodal negative cN0/pN0 N = 39	Occult metastastic cN0/pN+ N = 8	cN+/pN+ vs. cN0/pN0 P value	cN0/pN0 vs. cN0/pN+ P value		cN+/pN+ vs. cN0/pN+ P value
**Age (years)**								
*Median (IQR)*	62 (53–72)	59 (48–63.75)	66 (53–74)	63 (58.25–78.25)	0.039*	0.86	0.54	
**Gender**								
*Male*	48 (67.6%)	19 (79.8%)	25 (64.1%)	4 (50.0%)	0.25	0.69	0.17	
*Female*	23 (32.4%)	5 (20.8%)	14 (35.9%)	4 (50.0%)				
**Smoking**								
*Yes*	41 (57.7%)	18 (75.0%)	20 (51.3%)	3 (37.5%)	0.1	0.69	0.088	
*No*	30 (42.3%)	6 (25.0%)	19 (48.7%)	5 (62.5%)				
**Alcohol**								
*Yes*	29 (40.8%)	12 (50.0%)	14 (35.9%)	3 (37.5%)	0.17	0.68	0.69	
*No*	42 (59.2%)	12 (50.0%)	25 (64.1%)	5 (62.5%)				
**Tumor subsite**								
*Oral tongue*	38 (53.5%)	12 (50.0%)	21 (53.8%)	5 (62.5%)	0.48	0.88	0.47	
*Floor of mouth*	26 (36.6%)	11 (45.8%)	13 (33.3%)	2 (25.0%)				
*Other*	7 (9.8%)	1 (4.2%)	5 (12.9%)	1 (12.5%)				
**T category**								
*pT1*	13 (18.3%)	6 (25.0%)	7 (18%)	0 (0%)	0.13	0.097	0.34	
*pT2*	31 (43.7%)	7 (29.2%)	21 (53.8%)	3 (37.5%)				
*pT3*	11 (15.5%)	4 (16.7%)	4 (10.3%)	3 (37.5%)				
*pT4*	16 (22.5%)	7 (29.2%)	7 (17.9%)	2 (25.0%)				
**N category**								
*cN+/pN+*	24 (33.8%)	24 (100%)			<0.001*	<0.001*	<0.001*	
*cN0/pN0*	39 (54.9%)		39 (100%)					
*cN0/pN+*	8 (11.3%)			8 (100%)				
**Dissected nodes**								
*Mean (SEM)*	25.9 (±1.7)	30.9 (±2.6)	23.0 (±2.6)	24.3 (±2.8)	0.024*	0.88	0.039*	
**Positive nodes**								
*Mean (SEM)*	1.16 (±0.21)	2.8 (±0.41)	0	2 (±0.38)	<0.001*	<0.001*	0.62	
**Largest node (mm)**								
*Mean (SEM)*	7.38 (±1.57)	20.3 (±3.1)	0	4.1 (±0.76)	<0.001*	<0.001*	<0.003*	

*Statistically significant (*p* value < 0.05). Iqr: interquartile range. Sem: standard error of mean.

Median test for comparison of continuous variables. Fisher exact test for comparison of categorical variables.

### Association of higher SUV_max_ of primary tumor with occult metastatic disease in univariable analysis

**Figure 1:**
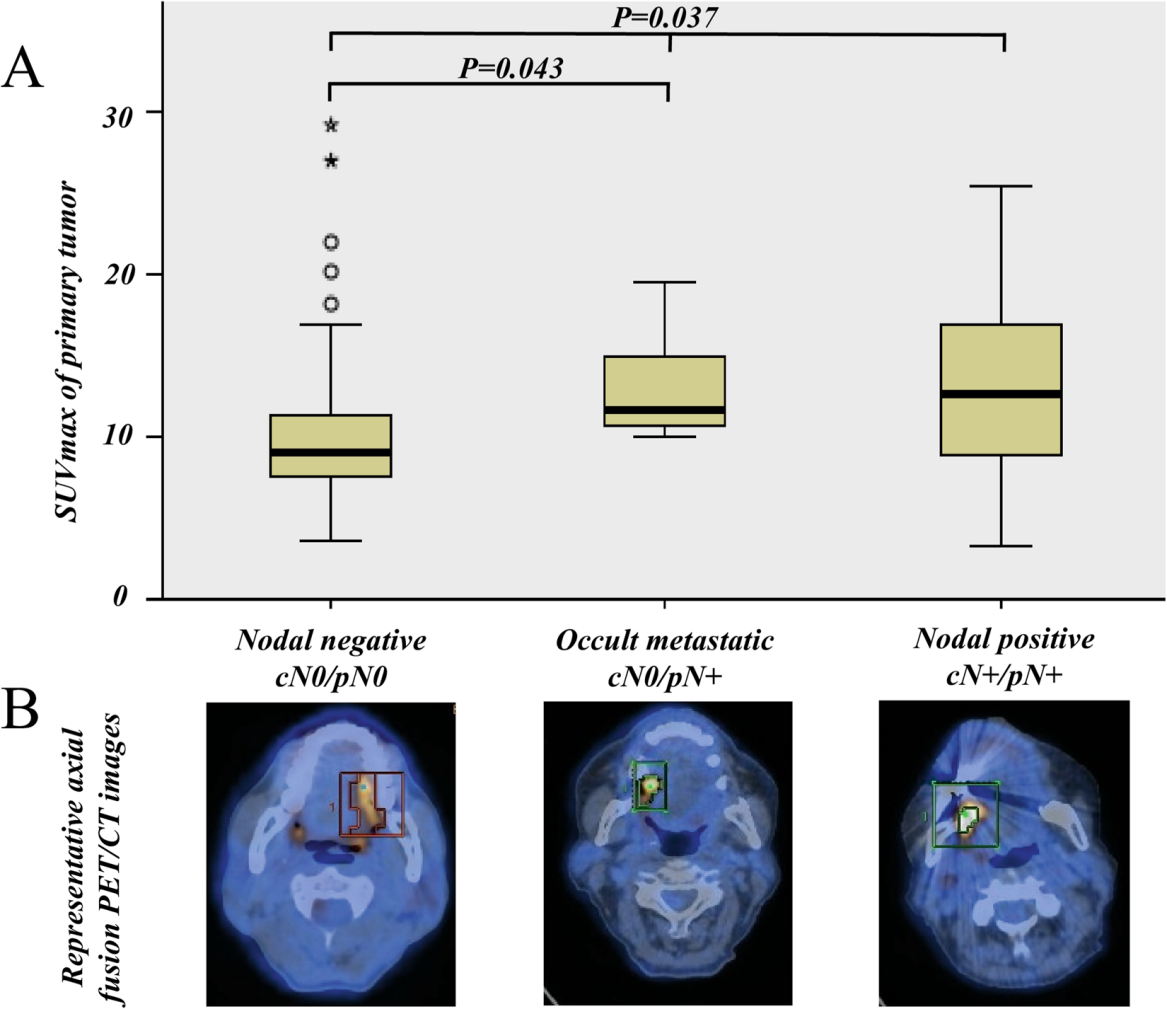
Comparison of medians across study groups showing significantly lower SUV_max_ in nodal negative cases than in occult metastatic and nodal positive cases (Independent samples median test, *P* = 0.037), as represented with (**A**) box-plot with median and interquartile range (IQR) and (**B**) by representative axial FDG-PET/CT images. When comparing the nodal negative group (cN0/pN0) to the occult metastatic group (cN0/pN+) alone, the difference remained significant (2 groups comparison, independent samples median test, *P* = 0.043).

SUV_max_ of the primary tumor was compared among study groups. In the nodal negative group (cN0/pN0), median SUV_max_ was 9.0 (IQR 7.4–13.9), while it was 11.4 (IQR 9.9–15.7) in the occult metastatic group (cN0/pN+) and 12.5 (IQR 8.5–16.9) in the nodal positive group (cN+/pN+). The difference was statistically significant (3 groups comparison, independent samples median test, *P* = 0.037) (Fig. 1). When comparing the nodal negative group (cN0/pN0) to the occult metastatic group (cN0/pN+) alone, the difference remained significant (2 groups comparison, independent samples median test, *P* = 0.043)

When comparing MTV, no statistical difference was found (independent samples median test, *P* = 0.26). For TLG, nodal positive group (cN+/pN+) and occult metastatic group (cN0/pN+) showed a trend towards higher values than the nodal negative group (cN0/pN0) (independent samples median test, *P* = 0.082).

### SUV_max_ ≥ 9.5 of primary tumor predicts occult metastatic disease in multivariable analysis

**Figure 2:**
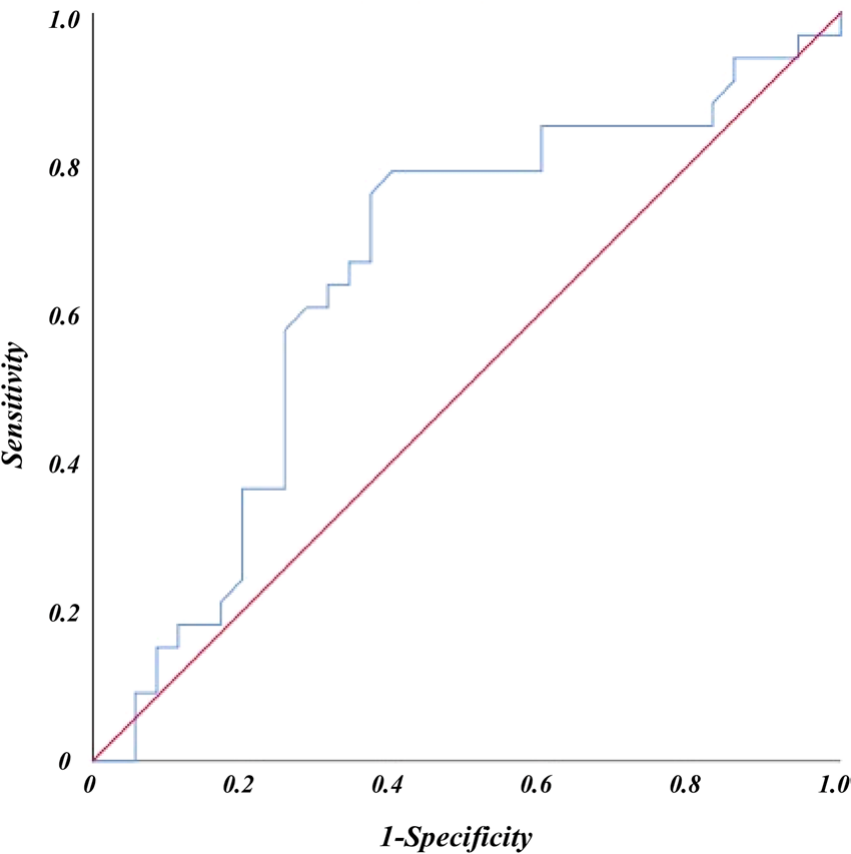
Receiver operating characteristic (ROC) curve analysis of lymph node metastasis prediction according to SUVmax of primary tumor. The area under the ROC curve was 0.651 (95% CI = 0.516–0.786, *P* = 0.032) and 9.5 was determined as best potential cutoff value for comparison. The sensitivity and specificity for SUVmax = 9.5 were 78.8% and 62.9%, respectively.

Receiver operating characteristic (ROC) analysis showed that the best potential cutoff value for SUVmax of primary tumor was 9.5 in prediction of occult metastatic disease (Fig. 2). The sensitivity and specificity for SUVmax = 9.5 were 78.8% and 62.9%, respectively.

To adjust to potential confounders, we built a binary logistic regression model including all relevant variables and included them in a multivariable model (Table 2). The only independent predictor of occult metastatic disease for cN0 patients was “SUV_max_ ≥ 9.5” (*P* = 0.028). This indicates that primary tumors with an SUV_max_ ≥ 9.5 have a statistically significant higher risk of harbouring occult lymph node metastasis.

**Table 2:** logistic regression model for prediction of occult lymph node metastasis^§^.

Variable	P value
**Gender**	
*Male vs. Female*	0.160
**Smoking**	
*Yes vs. No*	0.336
**Alcohol**	
*Yes vs*. *No*	0.213
**pt Category**	
*T1 vs*. *T2 vs*. *T3 vs*. *T4*	0.273
**SUVmax**	
≥*9.5 vs*. <*9.5*	0.028*

^§^Stepwise multivariable model.

^*^Statistically significant (*p* value < 0.05).

### Higher SUV_max_ of primary tumor predicts more extensive nodal involvement

**Figure 3:**
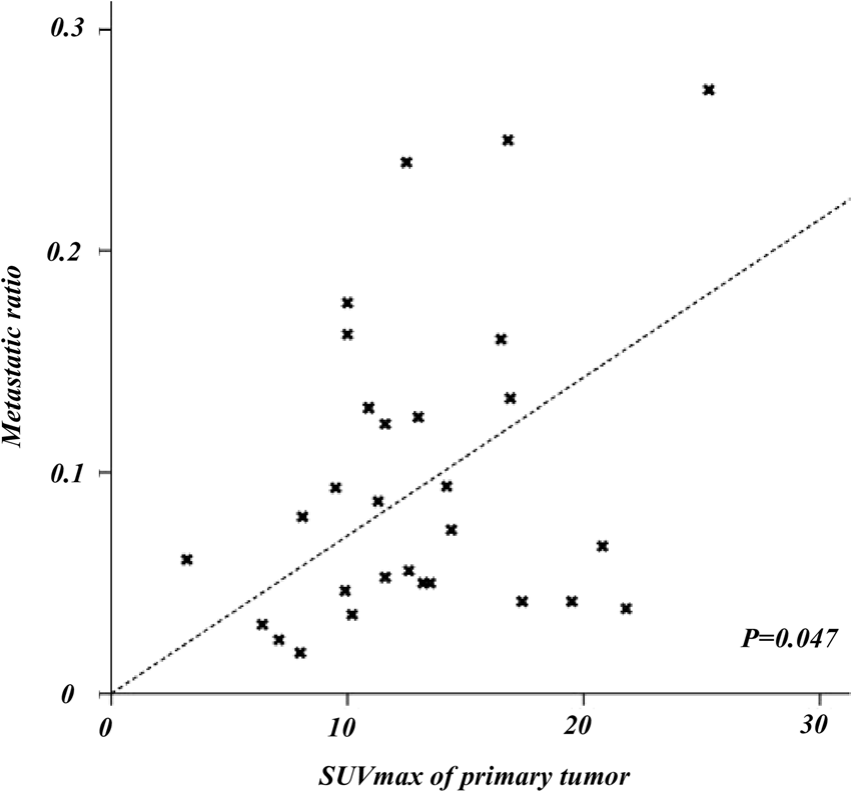
Correlation between SUV_max_ of primary tumor and metastatic disease. A higher SUV_max_ was associated with a higher metastatic ratio (Spearman rho: 0.243, *P* = 0.047).

In a third step, we investigated the impact of the SUV_max_ of the primary on nodal disease using quantitative outcome variables. A tumor showing a greater SUV_max_ was significantly associated with higher metastatic ratio (Spearman rho test, *P* = 0.047; Fig. 3). SUV_max_ of the primary tumor did not correlate with the diameter of the largest node (Spearman rho test, *P* = 0.12). For TLG and MTV, no statistical relation with extension of nodal involvement was seen (*P* > 0.05).

### Impact of SUV_max_ of primary tumor on oncological outcomes

There were 12 (16.9%) local recurrences in the cohort, whereas 13 (18.3%) patients had regional recurrence, and 7 (9.8%) had distant metastasis. Twelve (16.9%) patients experienced tumor-associated deaths, while there was a total of 20 (28.2%) deaths in the cohort.

The SUV_max_ of the primary tumor was not a predictor of overall survival (Log rank test, *P* = 0.98), disease-specific survival (Log rank test, *P* = 0.26), distant metastasis-free survival (Log rank test, *P* = 0.42), or regional recurrence-free survival (Log rank test, *P* = 0.20).

**Figure 4:**
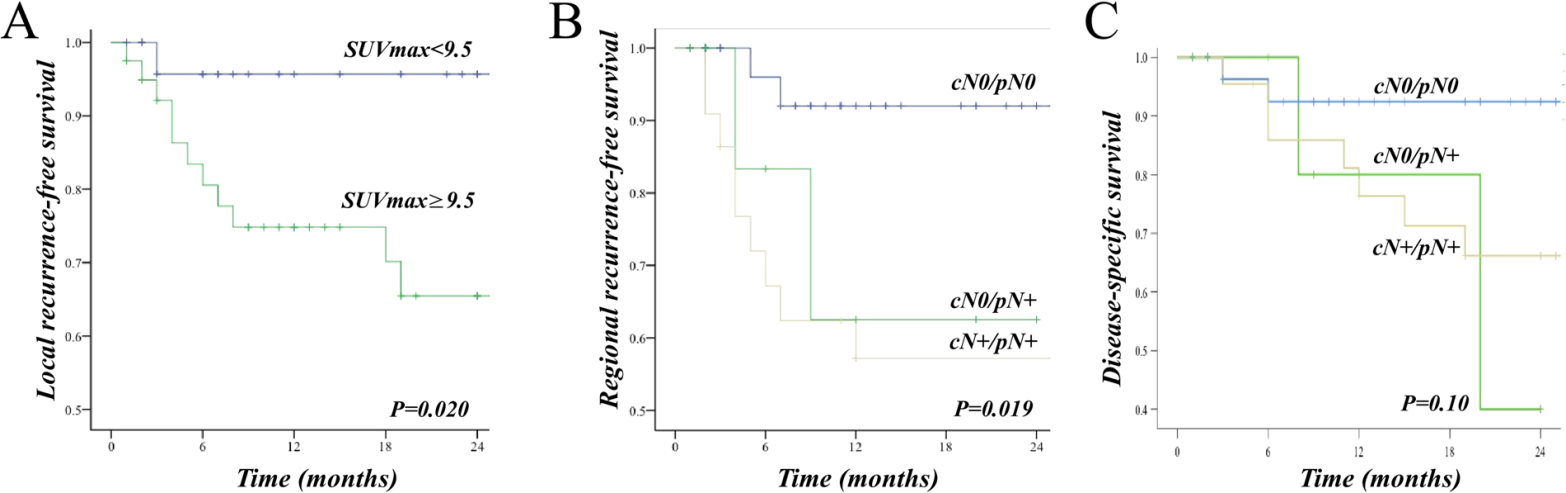
(**A**) Kaplan-Meier analysis showing relative survival according to SUV_max_ of primary tumor. High SUV_max_ predicted poorer local recurrence-free survival (Log rank test, *P* = 0.020). (**B**) Kaplan-Meier analysis showing regional recurrence-free survival according to study group (cN0/pN0, cN0/pN+, cN+/pN+) (Log rank test, *P* = 0.019). (**C**) Kaplan-Meier analysis showing disease-specific survival according to study group ((cN0/pN0, cN0/pN+, cN+/pN+) (Log rank test, *P* = 0.10).

Primary tumors with SUV_max_ ≥ 9.5 had a significantly higher risk of local recurrence (Log rank test, *P* = 0.020) (Fig. 4A). Similarly, tumors with TLG ≥ 28.4 had a significantly higher risk of local recurrence (Log rank test, *P* = 0.011, not shown), while the regional recurrence-free survival, distant metastasis-free survival, disease-specific and overall survival did not depend on TLG and/or MTV (Log rank test, *P* > 0.05).

When comparing outcome according to study group, there was no difference in local recurrence-free survival between study groups (Log rank test, *P* = 0.148). Regional recurrence-free survival was worse in the nodal positive (cN+/pN+) and occult metastatic (cN0/pN+) groups than in the cN0/pN0 group (Log rank test, *P* = 0.019) (Fig. 4B), while there was a trend towards worse disease-specific survival for nodal positive groups (Log rank test; *P* = 0.10) (Fig. 4C). For overall survival and distant metastasis-free survival, the differences were not significant (Log rank test, *P* = 0.52 and *P* = 0.17).

## Discussion

This study evaluates in a cohort of OSCC the predictability of occult lymph node metastasis by PET/CT metabolic parameters of the primary tumor. Tumors with higher SUV_max_ showed higher propensity to nodal metastasis in univariable and multivariable analysis and higher metastatic ratio. These tumors also showed poorer local recurrence free-survival. For TLG (but not for MTV), we observed similar correlations to those observed for SUV_max_.

In head and neck cancers, all three metabolic parameters SUV_max_, MTV and TLG have been described as predictor of outcome, as reviewed recently by Castelli *et al*.^23^, mainly in the context of response to primary chemoradiation^23^. Although SUV_max_ is merely a single-voxel value representing the most intense FDG uptake of the tumor, it seems that among all metabolic parameters, it is the one showing the highest predictive diagnostic accuracy^23^, in carcinomas and/or other tumor entities such as lymphomas^24^. Due to the simplicity of its measurement, it is also of practical use in daily clinical routine. In our study, we showed that the SUV_max_ and to a lesser extent TLG were predictors of occult nodal metastasis and outcome. To the best of our knowledge, we are the first to show this correlation in OSCC. A few similar studies have been performed for lung cancer^17,18,25^. These also showed that occult lymph node metastases could be predicted using metabolic parameters of the primary tumor^12,13,20^.

The cut-off set in these lung cancer studies appeared to be different according to the histology of the primary tumor, with squamous cell carcinoma showing a higher SUV_max_ than adenocarcinoma^25^. For squamous cell carcinoma of the lung, the cut-off was SUV_max_ ≥ 8.8^17^, which is comparable to our study.

For cN0 oral cancer, if an elective neck dissection is preferred over a sentinel lymph node biopsy, a neck dissection levels I-III (formerly called “supraomohyoid neck dissection”) will usually be performed^5^. However, in nodal positive cases, a therapeutic neck dissection is mandatory. The neck is addressed consequently in a more comprehensive fashion. Such cases are classically treated with radical or modified radical neck dissection, although a selective neck dissection of Level I-IV may be performed as well^5^. This is reflected in our study by the total number of dissected lymph nodes in the final histopathology specimen, which was significantly higher in the nodal positive group (therapeutic neck dissection) than in the occult metastatic group (elective neck dissection) (Table 1).

Although the size of nodal metastases was smaller in the occult metastatic group, the regional recurrence-free survival was similar to the nodal positive group (Fig. 4C). This data, together with the poorer local recurrence-free survival for tumors, may encourage surgeons towards a more aggressive management in patients with a high SUV_max_ of the primary tumor. Whether this would result in improved locoregional recurrence-free survival is however beyond the scope of this study and remains hypothetical.

However, our study has some limitations: First, the design was retrospective. Second, PET scans were acquired on different scanners, nevertheless, the SUV_max_ and other PET parameters are standardized measures. Third, we had a relative low number of patients, in particular in the occult metastatic group (cN0/pN+), which renders the statistical analysis harder and might lead to beta error^22^. Further, owing to the small size of the study population, we did not perform a sub-analysis depending on the HPV status or on the anatomical subsite of the primary tumor within the oral cavity, but in contrast to oropharyngeal cancers, the percentage of HPV-positive tumors in the oral cavity is significantly lower and therefore less confounding.

In conclusion, we show the importance of SUV_max_ of primary tumor in prediction of occult nodal metastases in oral cancer. We believe this simple information provided by FDG-PET can, in combination with other parameters, be a useful pre-therapeutic indicator impacting surgical management of patients.
